# PILOTING AN INTERPROFESSIONAL GERIATRICS COURSE FOCUSED ON SYSTEMS-BASED PRACTICE

**DOI:** 10.1093/geroni/igz038.545

**Published:** 2019-11-08

**Authors:** Jennifer J Severance, Sarah Ross

**Affiliations:** University of North Texas Health Science Center, Fort Worth, Texas, United States

## Abstract

Health professionals must be able to work together in the care of older adults to provide competent, value-based care within dynamic healthcare systems. Previous studies cite the need for health professions education addressing systems-based competencies and the interconnected components of health and aging services. With an aim to provide a foundation for systems-based interprofessional practice in the care of older adults, an academic medical center piloted a competency-based curriculum introducing different settings of care for older adults and exploring special topics in geriatric patient care and gerontology. Applying a framework of health and aging services, interprofessional faculty developed a curriculum delivered over six one-hour sessions during the Spring 2019 semester. Session topics included dementia care, end-of-life care, medication safety, activity, and financial and regulatory considerations across the continuum of care. Teaching strategies included didactics, case-based discussions, online reflection questions, shadowing of geriatric encounters in clinic and long-term care settings, and student presentations of a patient case. Post-session evaluation surveys were administered electronically to measure knowledge, skills and confidence using Likert-scale and open-ended questions. Eleven health professions students (medical [n=6], physician assistant [n=3], physical therapy [n=2]) participated. Preliminary findings show improved knowledge about healthcare and community-based options for older adults, value-based care, and the driving forces affecting health care delivery. Analysis of qualitative feedback identified key themes related to teaching methods, course materials, and course improvement opportunities. Interprofessional faculty collaboration can lead to quality health professions student training and increase competency in systems-based practice in the care of older adults.

